# A case of intraoperative arrest & mobile ECMO

**DOI:** 10.1051/ject/2025003

**Published:** 2025-03-07

**Authors:** Rodrigo Alejandro Díaz Gómez, Catalina Alvarado Neves, Carmen Gloria Karlezi de la Fuente, Gabriela Cecilia Bejarano Alva, Dafna Garcia Gomez, Luisa Fernanda Rodas García

**Affiliations:** 1 Clinica Red Salud Santiago Av. Libertador Bernardo O'Higgins 4850, Estación Central Santiago Región Metropolitana de Santiago Chile; 2 Hospital Regional de Moyobamba Av. Grau Cuadra 4, Barrio Calvario- – Moyobamba – Moyobamba San Martín Perú; 3 Fundacion Cardiovascular de Colombia Urbanización El Bosque. Floridablanca Santander Colombia; 4 Instituto Guatemalteco de Seguridad Social 7a. avenida 22-72, zona 1 Ciudad de Guatemala

**Keywords:** ECMO, ECPR, Resuscitation, Pulmonary embolism

## Abstract

Over the past two decades, extracorporeal membrane oxygenation (ECMO) has been increasingly used to support critical patients with cardiac and respiratory failure who fail to respond to conventional management. In refractory cardiac arrest, ECMO can restore perfusion in patients who meet specific criteria designed to maximize survival benefit and good neurological outcomes. In recent literature, there is no report of mobile ECMO in a case of prolonged cardiac arrest with direct cardiac massage. We describe our experience with a 34-year-old man with multiple traumatic injuries following a motor vehicle collision. He was treated in a trauma center hospital in the same city as our center. He was initially in stable condition (spontaneous ventilation with FiO2 0.21, no vasoactive drugs, Glasgow 15, no acute kidney injury or other organ dysfunction). One week after admission, a retained left hemopneumothorax required surgical intervention, as previous drainage was ineffective. Computed tomography imaging was also concerning for parencyhmal injury by the thoracotomy tube. Intraoperatively, when the patient was placed in lateral position, he experienced cardiac arrest, presumed to be secondary to pulmonary embolism. After 18 min, we were asked to rescue this patient with ECMO, as he had no contraindications to support. After 81 min of advanced life support, including direct cardiac massage, return of spontaneous circulation was achieved seconds after ECMO was initiated. He was then transported to our hospital. The patient achieved a favorable neurological outcome (Glasgow Coma Scale score of 15 at 24 h) and was discharged after a 2 month stay. This case highlights the potential benefits of prolonged cardiopulmonary resuscitation and ECMO in patients with refractory in-hospital cardiac arrest. In this case, proper ACLS and CPR allowed time for mobile ECMO support to be initiated from a remote center.

## Overview

Even in cases occurring within hospitals, survival rates following extended cardiac arrest are low. A multicenter study examining 348,996 intra-hospital cardiac arrest (IHCA) incidents discovered that less than 2.5% of patients experienced favorable outcomes after receiving CPR for over 40 min in shockable rhythm scenarios [1].

IHCA in the operating room is uncommon, occurring at a rate of 3 per 10,000 anesthetics in the UK. However, if it persists for more than 5–10 min, managing the situation becomes significantly more complex and often results in a poorer prognosis [2].

To enhance survival rates and minimize neurological impairment, it is crucial to identify the issue, intervene promptly, systematically address reversible causes, and, in prolonged cases, evaluate the use of cardiopulmonary bypass (CPB) or ECMO in some situations, as recommended by Stanford Cognitive Aids for Perioperative Crisis guidelines [3]. However, what actions can be taken if spontaneous circulation does not resume after several minutes and ECMO or CPB are unavailable?

In the United Kingdom, just 9% of centers involved in analyzing intra-operative cardiac arrest (as part of the 7th National Audit Project by the Royal College of Anaesthetists) had ECMO available. Moreover, only 9 out of 548 patients received ECMO support [2]. A potential solution could be to activate a mobile ECMO team. A 2023 review comparing ECPR with manual or mechanical CPR during cardiac arrest found 3 trials, 27 observational studies, and 6 cost-effectiveness analyses, indicating the potential benefits of ECPR [4].

The survival advantage of open-chest CPR (OCCPR) compared to closed-chest CPR (CCCPR) remains uncertain. CCCPR is the standard practice [5], however, European guidelines advise considering open-chest cardiac massage during a peri-operative cardiac arrest if ROSC cannot be achieved through closed compressions and ECPR is not available [6].

Trauma does not rule out the use of ECMO, which has been utilized more frequently in this situation. Advances in anticoagulation and technology strive to reduce associated complications. While formal guidelines have yet to be established, clinicians recognize its potential life-saving benefits for critically injured patients [7].

At the moment of establishing a “system” for a situation like this, Germany’s Düsseldorf ECLS Network offers advanced mechanical support for critically ill patients at multiple centers. Of the 160 patients, 102 received ECMO during cardiac arrest, with a 34% survival rate to discharge [8]. The rendezvous model strategy aims to enhance patient outcomes by initiating ECMO support earlier in the prehospital environment, utilizing a central hub that serves multiple centers, and then back to a central ECMO hospital once cannulated. This approach necessitates extensive planning, logistics, and collaboration among various institutions [9]. A cannulation team in Southern California successfully transported patients requiring ECMO support. This included several ECPR cases involving drug overdoses and refractory arrhythmias [10].

## Description

A 34-year-old male patient with no significant past medical history presented to the emergency department of a public hospital in Chile, shortly after a traffic collision. The patient presented with blunt chest trauma, including left hemopneumothorax, multiple rib and sternal fractures, thoracic vertebral fractures (T1; T4-T8), an open fracture of the left wrist and a fracture of the left elbow. Additionally, blunt abdominal trauma led to a splenic injury. Consequently, DVT prophylaxis was not indicated.

Upon hospital arrival, a pleurostomy was performed but was found to be ineffective, with impaction into the lung parenchyma and no air or blood was drained. Due to reasons beyond the medical team’s control, surgical removal of this tube and drainage of hemopneumothorax was planned seven days after admission. The patient at that time was in good general condition, hemodynamically stable with no vasoactive drugs, spontaneous ventilation, with no O_2_ required.

At the beginning of the surgery, the patient unexpectedly experienced loss of the capnography waveform. While investigating the cause, the patient developed ventricular fibrillation (VF), followed by asystole. The surgical procedure was immediately halted and the patient was placed in the supine position. Following confirmation of pulselessness, CPR was initiated according to established protocols. A thoracotomy was performed to rule out cardiac tamponade, and internal cardiac massage was initiated, followed by direct defibrillation. The rhythm converted to pulseless electrical activity and kept mainly that pattern.

Echocardiography revealed enlargement of the right heart chamber, suggesting pulmonary thromboembolism. The clinical symptoms further supported this diagnosis.

**Figure 1:**
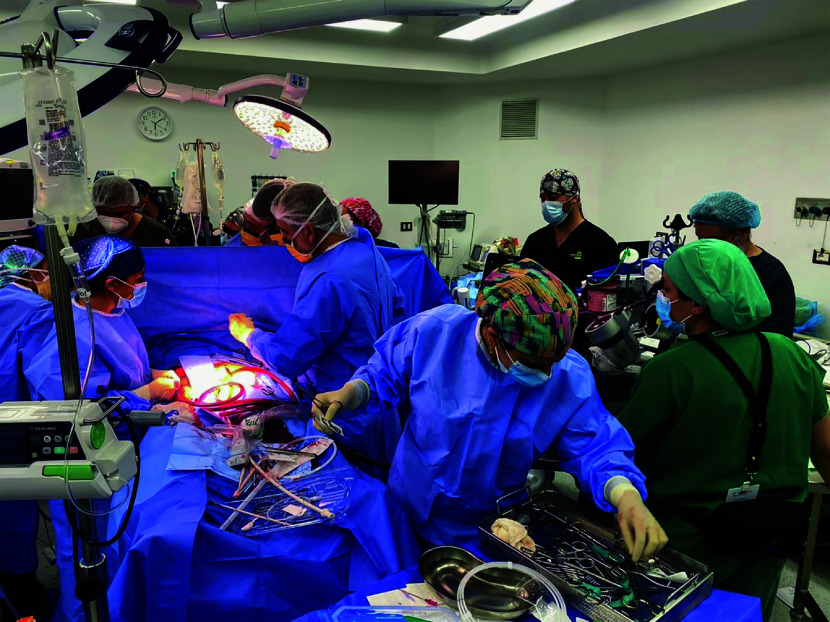
ECPR CANNULATION in the operating room the patient presented the arrest and the ECMO team came in, reevaluated the situation and proceeded to go on with cannulation.

Due to prolonged unresponsive cardiac arrest and the absence of ROSC, the nearest ECMO Team was contacted after 18 min of cardiac arrest. Arterial blood gas control showed metabolic acidosis (pH 7.12). Considering that arrest was witnessed, initial VF rhythm, patient′s age, and no identifiable irreversible condition, following unsuccessful CPR, ECMO support was initiated after 81 min of uninterrupted resuscitation measures. Cannulation was performed via the right femoral vein (29 Fr cannula) and the left femoral artery (17 Fr cannula) with a distal reperfusion catheter (7 Fr) (Fig. 1). An EUROSETS ECMO Adult polymethylpentene fiber oxygenator (Eurosets, Medolla, Italy) and a ROTAFLOW I Centrifugal pump (Getinge, Gothenburg, Sweden) were used. The patient was subsequently transferred to our ECMO team center.

Mild therapeutic hypothermia was administered for 16 h and was later reversed. Upon confirmation of the neurological integrity, the patient was extubated.

**Figure 2:**
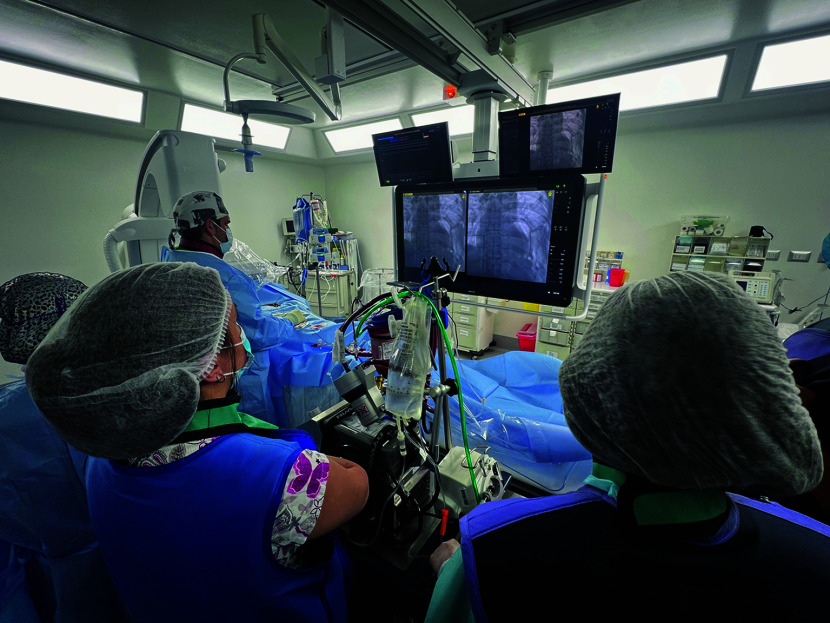
Pulmonary angiography (24 h after Cardiac arrest), no pulmonary emboli was identified.

After 24 h of ECMO support, pulmonary angiography excluded a massive pulmonary embolism (Fig. 2). However, segmental thrombosis in the right internal saphenous vein was identified on Doppler ultrasound.

**Figure 3:**
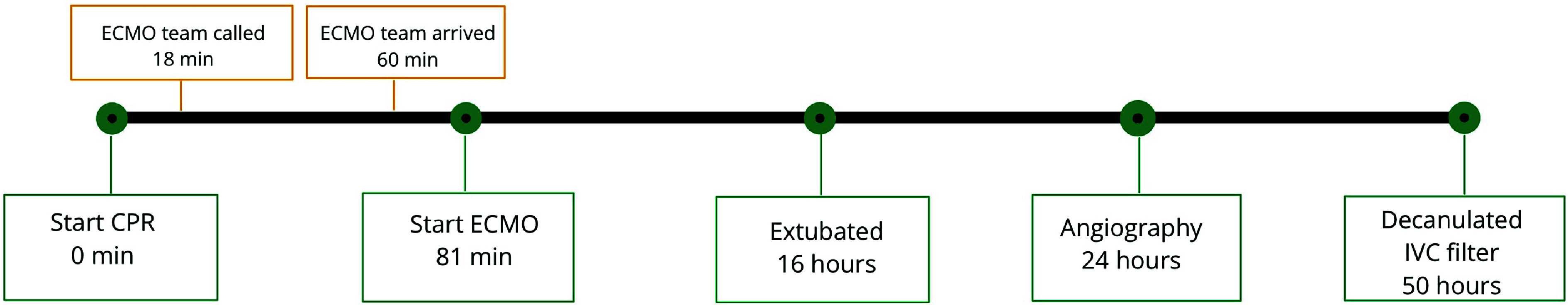
Case timeline.

ECMO support was successfully weaned after 50 h, and an inferior vena cava filter was placed to prevent further embolization (Fig. 3).

The patient was discharged from the hospital after 64 days of stance with no neurological sequelae and physical therapy to recover functional capacity.

This case report highlights the unique combination of mobile extracorporeal cardiopulmonary resuscitation (ECPR), direct cardiac massage and prolonged resuscitation. Successful ECPR requires a well-coordinated multidisciplinary team, optimal patient selection, adequate resources, interinstitutional collaboration and timely intervention.

## Comment

In cases of in-hospital cardiac arrest (IHCA), the decision to withdraw resuscitation should not rely solely on the elapsed time. Several factors warrant consideration, including patient age, reversibility of cardiac arrest, initial rhythm, underlying medical conditions, resuscitation quality, and CPR response.

In this specific case, prompt and effective resuscitation efforts coupled with early consideration of ECMO, timely cannulation and transfer to a specialized center contributed to a successful outcome.

This case report underscores the potential life – saving benefits of a rapid and aggressive multidisciplinary approach, including mobile ECPR and prolonged direct cardiac massage in patients with refractory cardiac arrest, highlighting the uniqueness of this rare combination. While further research is needed to validate these findings, our experience suggests that strategy may offer hope and expand the therapeutic window for patients with otherwise unsurvivable cardiac arrest in a center without an ECMO program.

## Data Availability

All available data are incorporated into the article.
